# A short‐term in vivo model for Merkel Cell Carcinoma

**DOI:** 10.1111/exd.13529

**Published:** 2018-03-26

**Authors:** Vishwanath Kumble Bhat, Corinna Krump, Eva Bernhart, Jürgen C. Becker, Wolfgang Sattler, Nassim Ghaffari‐Tabrizi‐Wizsy

**Affiliations:** ^1^ Institute of Molecular Biology and Biochemistry Medical University of Graz Graz Austria; ^2^ Institute of Pathophysiology and Immunology Medical University of Graz Graz Austria; ^3^ Department for Translational Skin Cancer Research & Department of Dermatology University Hospital Essen University of Duisburg‐Essen Essen Germany; ^4^ German Cancer Consortium (DKTK) Partner Site Essen/Düsseldorf Düsseldorf Germany

**Keywords:** Chorioallantoic membrane assay, MCPyV‐LT antigen, Merkel cell carcinoma

## Abstract

In vivo tumor models are essential for studying the biology of cancer, identifying tumor targets and evaluating antitumor drugs. Considering the request for the minimisation of animal experiments and following the “3R”‐rule (“replacement,” “refinement,” “reduction”), it has become crucial to develop alternative experimental models in cancer biology. Several studies have already described the avian chorioallantoic membrane (CAM) model as an alternative to rodents, suitable to investigate growth, progression and metastasis of various types of cancer. In the present work, we grafted three Merkel cell carcinoma (MCC) cell lines onto the avian CAM and monitored tumor growth and development of solid tumor nodules. Morphology of xenograft was characterised histologically and immunohistochemically. Our results demonstrate CAM assay as a useful tool to study MCC pathophysiology.

## 1 BACKGROUND

Merkel Cell Carcinoma (MCC) is a rare, highly aggressive neuroendocrine tumor of the skin with poor prognosis that typically occurs in elderly and immunosuppressed patients.^1^ The MCC is characterised by the presence of cytokeratin 20 (CK‐20) and neuroendocrine granules. The outcome of immune surveillance suggested viral carcinogenesis, which was indeed demonstrated in the majority of cases.^2^ UV radiation exposure is an additional epidemiologic risk factor for MCC.^3^ Due to the development of immune checkpoint inhibitors, a new therapeutic window opened for MCC patients.^4^, ^5^, ^6^ Recently, treatment with three humanized antibodies, namely avelumab, pembrolizumab and nivolumab targeting PD‐L1/PD‐1 pathway have shown durable responses in MCC patients, and avelumab has been approved by the FDA for the treatment of advanced MCC.^7^ Even though a panel of well characterised MCC cell lines is available,^8^, ^9^, ^10^ the use of these cells in 2D culture systems is of limited value for translation into clinical settings. Therefore, we aimed to establish an in vivo model for MCC using the CAM assay to investigate growth and proliferation properties of onplanted MCC tumors. The CAM model is a time‐ and cost‐effective drug screening system that was successfully used to characterise growth, proliferation and metastasis in a number of other cancer entities.^11^, ^12^, ^13^

## 2 QUESTION ASKED

We investigated whether the CAM system is suitable as a short‐term in vivo model for MCC to study tumor growth, proliferation and angiogenesis.

## 3 EXPERIMENTAL DESIGN

We grafted three MCPyV‐positive MCC cell lines (MKL‐1, PeTa, WaGa), that clearly differ in their phenotype and growth behaviour in cell culture in vitro, onto the CAM and monitored proliferation as well as development of solid tumor nodules and characterised their morphology as shown schematically in Figure S1. We performed three independent experiments with four onplants for each cell line.

## 4 RESULTS

**Figure 1:**
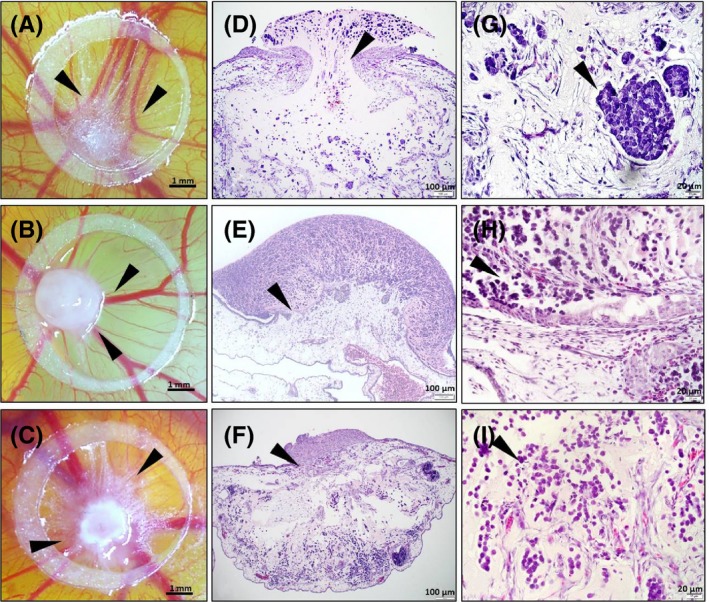
Morphological analysis of MCC cell lines on CAM. (A‐C) Ex ovo CAM assay: MCC cell lines formed solid tumors 5 d upon transplantation within the silicone ring on the CAM surface, and avian vessels developed radially towards the onplants (arrows), MKL‐1 (A), PeTa (B) and WaGa (C) (10× Magnification, bars equal 1 mm). (D‐F) Morphological analysis of haematoxylin/eosin stained sections revealed outgrowth of tumor cells from the primary onplant site into the surrounding CAM tissue thereby disrupting the CAM upper epithelium (arrows), MKL‐1 (D), PeTa (E) and WaGa (F) (100× Magnification, bars equal 100 μm). (GI) MCC cell lines form tumors composed of strands or nests of uniform, small round cells with marginal cytoplasm and round nucleus (arrows), MKL‐1 (G), PeTa (H), WaGa (I) (400× magnification, bars equal 20 μm)

All cell lines formed tumors within 3 days after transplantation; progression of tumor formation was monitored by photo‐documentation throughout the incubation period (Figure S2A). MKL‐1, PeTa and WaGa tumors revealed a reproducible growth pattern. MCC cells developed into solid nodules from day 3 after transplantation; vascularisation steadily progressed, and avian vessels developed radially towards the tumors (Figure 1A‐C, Figure S2A). On day 5 posttransplantation, the tumor area (mm2) was determined by ImageJ (Figure S2B), and the number of vessels surrounding the xenografts was counted manually (Figure S2C) according to Ribatti et al, 2006.^14^ We did not observe any significant difference within MCC cell lines with respect to tumor area or macroscopic blood vessels.

At day 5 post transplantation, xenografted MCC tumors were excised, fixed, paraffin embedded and stained for haematoxylin and eosin (H and E); for details, see supplementary material. The tumors showed strong interaction of MCC cells with the CAM mesenchyme and invasion of tumor cells from the primary onplant site into the surrounding CAM tissue, thereby disrupting the CAM upper epithelium (Figure 1D‐F arrows). The histological appearance of the tumors was similar to those of MCC, composed of strands or nests of uniform, small round cells with marginal cytoplasm and round nucleus (Figure 1G‐I, arrows).^15^

**Figure 2:**
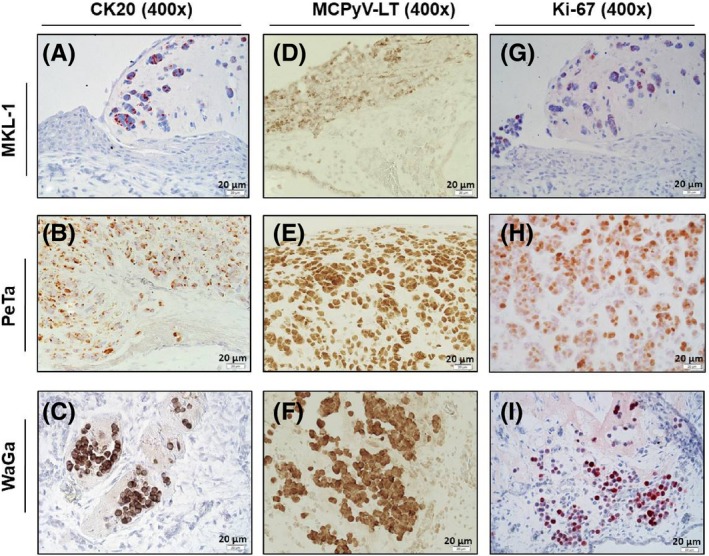
Immunohistochemical characterizations of xenografted MCC cell lines. (A‐C) MKL‐1, PeTa and WaGa MCC xenografts expressing MCC‐specific marker CK20 = cytokeratin 20 and (D‐F) MCPyV‐LT as well as the proliferation marker Ki‐67 (G‐I). (400× magnification, bars equal 20 μm)

Furthermore, using immunohistochemistry method (IHC), the sections were analysed for the expression of MCC marker CK‐20, MCPyV‐LT antigen and the proliferation marker Ki‐67 (Figure 2). Expression of specific neuroendocrine tumor markers such as chromogranin‐A (CGA) and synaptophysin (p‐38) are shown in Figure S3.^16^, ^17^

CK‐20 was present in all MCC xenografts in a typical dot‐like perinuclear staining pattern (Figure 2A‐C).^18^ The MCPyV‐LT antigen was detected in all three MCC cell lines. The IHC showed nuclear staining of LT antigen in MKL‐1 (Figure 2D) and cytoplasmic staining for PeTa and WaGa (Figure 2E‐F) due to the differences in truncating mutation in LT antigen.^19^ This staining of MCPyV LT antigen could be used as a valuable marker for drug screening of virus‐positive cells. The neuroendocrine marker CGA and p‐38 were strongly expressed in MKL‐1, PeTa and WaGa cells and allowed identification of single evaded tumor cells (Figure S3A‐F).

Ki‐67 was used to stain the proliferating cells distributed throughout the tumor mass. The positive Ki‐67 staining was seen in all three MCC cell lines indicating the tumor growth and proliferation (Figure 2G‐I). This will be useful to study the response of MCC cell lines to drugs under in vivo conditions.

## 5 CONCLUSION

We here demonstrated that the CAM system can be used as an experimental in vivo tool that reproduces tumor‐stroma interaction, angiogenesis and growth in MCC. Our data indicate that the CAM could represent a valuable preclinical model suitable to study MCC biology and drug response.

## CONFLICT OF INTEREST

The authors have declared no conflicting interests.

## AUTHOR CONTRIBUTIONS

VKB, CK and EB performed the research, NGTW designed the research study, JCB provided cell lines, NGTW contributed essential reagents and tools, VKB analysed the data. VKB, CK, JCB, WS and NGTW wrote the paper. VKB, EB, WS and NGTW revised the manuscript.

## Supporting information


**FIGURE S1** Schematic workflow of *ex ovo* CAM assay. Fertilized eggs were incubated for 3 days, the egg shell was then cracked into plastic dishes, following further incubation for 7 days. MCC cells were applied on vascular branches of the CAM and incubated for 3‐7 days. The CAM with the attached grafts was excised, followed by FFPE‐tissue embedding and sectioning. The tumour morphology was analysed by histology and immunostaining

**FIGURE S2** Photo‐documentation of growth behaviour of xenografted MCC cell lines. (A) MKL‐1 (upper panel), PeTa (middle panel) and WaGa (lower panel) were monitored for 5 days upon engraftment. Bars equal 1 mm. (B) Tumour area per CAM was measured using Image J software. (C) Angiogenesis was measured by counting macroscopic blood vessels (MBV) manually. Results were plotted as mean ± SD using GraphPad prism software. (N = 6 tumours). One‐way ANOVA was used for statistical analysis

**FIGURE S3** Immunohistochemical characterizations of xenografted MCC cell lines with neuroendocrine specific marker. (A‐F) All MCC cell lines express the neuroendocrine tumour specific markers synaptophysin (p38) and chromogranin A (100× and 400× magnification, scale bar = 100 µm and 20 μm respectively)
