# Supplementary figures and images for: A short‐term in vivo model for Merkel Cell Carcinoma
Source: Exp Dermatol. 2018 Mar 26;27(6):684–7. doi: 10.1111/exd.13529 (PMC6175323; doi:10.1111/exd.13529)

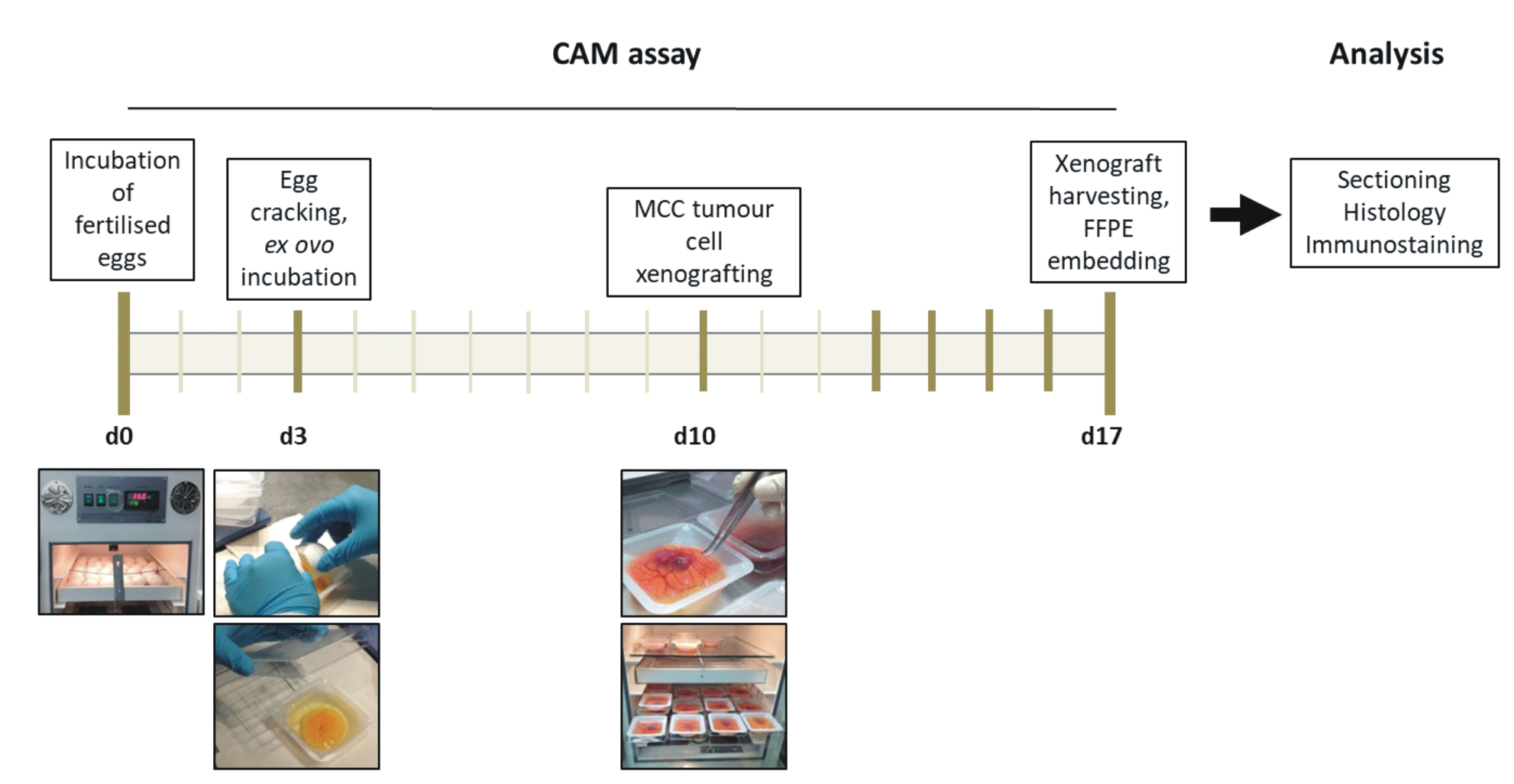

Supplement: Supplementary file 1 — FIGURE S1 Schematic workflow of ex ovo CAM assay. Fertilized eggs were incubated for 3 days, the egg shell was then cracked into plastic dishes, following further incubation for 7 days. MCC cells were applied on vascular branches of the CAM and incubated for 3‐7 days. The CAM with the attached grafts was excised, followed by FFPE‐tissue embedding and sectioning. The tumour morphology was analysed by histology and immunostaining [file EXD-27-684-s001.TIF]

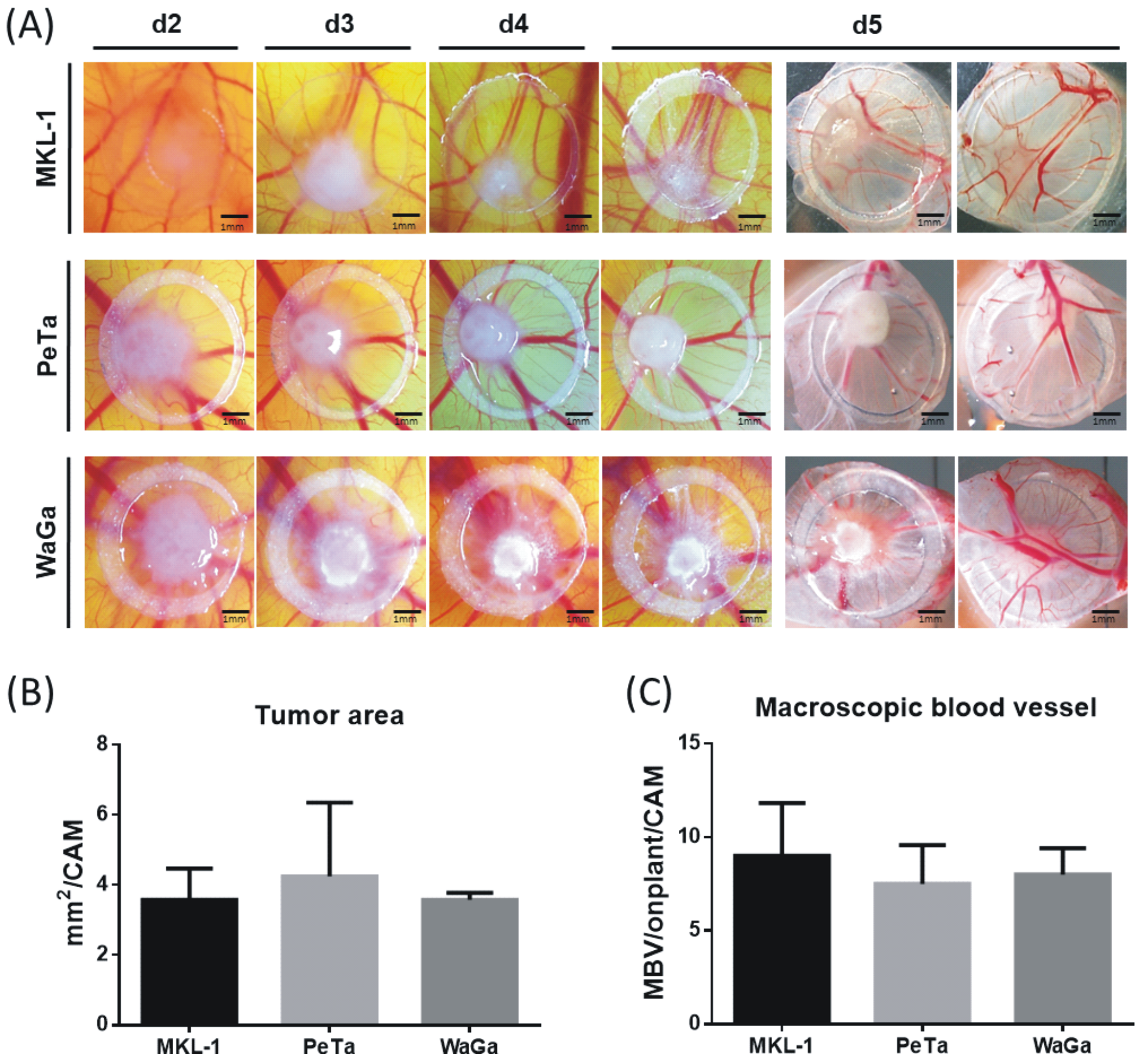

Supplement: Supplementary file 2 — FIGURE S2 Photo‐documentation of growth behaviour of xenografted MCC cell lines. (A) MKL‐1 (upper panel), PeTa (middle panel) and WaGa (lower panel) were monitored for 5 days upon engraftment. Bars equal 1 mm. (B) Tumour area per CAM was measured using Image J software. (C) Angiogenesis was measured by counting macroscopic blood vessels (MBV) manually. Results were plotted as mean ± SD using GraphPad prism software. (N = 6 tumours). One‐way ANOVA was used for statistical analysis [file EXD-27-684-s002.TIF]

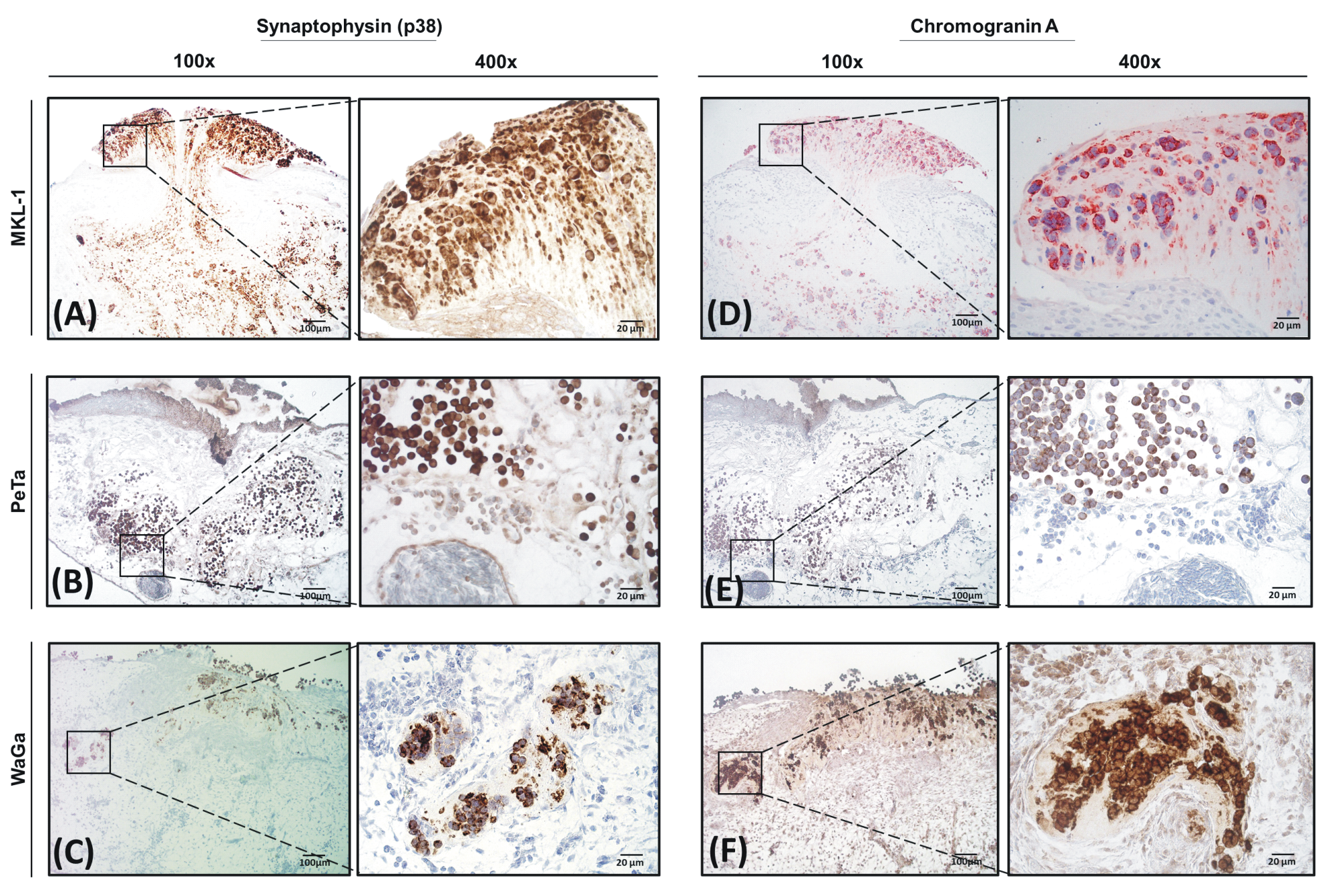

Supplement: Supplementary file 3 — FIGURE S3 Immunohistochemical characterizations of xenografted MCC cell lines with neuroendocrine specific marker. (A‐F) All MCC cell lines express the neuroendocrine tumour specific markers synaptophysin (p38) and chromogranin A (100× and 400× magnification, scale bar = 100 µm and 20 μm respectively) [file EXD-27-684-s003.TIF]
