# Supplementary material for: A short‐term in vivo model for Merkel Cell Carcinoma
Source: Exp Dermatol. 2018 Mar 26;27(6):684–7. doi: 10.1111/exd.13529 (PMC6175323; doi:10.1111/exd.13529)
Supplement: Supplementary file 4 [file EXD-27-684-s004.docx]

**Supplementary Material**

**Materials and Methods**

Cell Culture

Human Merkel Cell Carcinoma cell lines (MKL-1, PeTa, WaGa) were maintained as suspension cultures. MKL-1 and PeTa cells grow in aggregates forming irregular loose spheroid, while WaGa grow in single cell suspension. The cells were grown in a humidified incubator at 5 % CO_2_ and 37 °C in RPMI-1640 (Sigma-Aldrich) supplemented with 10 % FBS (Sigma-Aldrich) and 1 % penicillin/streptomycin (Biochrom). Trypsin/EDTA-solution (Biochrom) was used to detach spheroids.

CAM xenografts

*Ex ovo* CAM assays were performed as described by Deryugina & Quigley.[S1] In brief, fertilized eggs of white leghorn chicken from a local commercial hatchery were incubated for 3 days (37.6 °C, 50 % humidity) lying horizontally with continuous rotation. The egg shell was cracked at its centre perpendicular to the long axis of the egg by use of a mechanical fret saw. The content was placed into plastic dishes (sterilized with EtOH and subsequent UV-irradiation) for further incubation for a maximum of 7 days. MCC cells (WaGa, PeTa, MKL-1) were applied in volumes of 20 µL in a 1:1 mixture with Matrigel (BD Matrigel^TM^ Basement Membrane Mix) (10^6^cells/onplant) within silicone rings (5mm diameter) applied on vascular branches of the CAM. Progression of tumour development of cell xenografts was monitored by photo-documentation (Olympus SZX16) throughout the incubation period, 3-7 days post-transplantation. On day 15 of the embryo (corresponding to day 5 post transplantation), the tumours were photographed and tumour area was quantified using ImageJ software and blood vessels were counted manually.[S2, S3] The CAM with the attached grafts was removed by a square cut and the host chick embryo was killed by decapitation. Samples were washed and fixed with 4 % paraformaldehyde (16 h, room temperature) followed by FFPE-tissue embedding.

Histology

Paraffin-embedded samples were cut and 5µm sections were stained with haematoxylin/eosin or analysed immunohistochemically according to protocol (Mouse and Rabbit specific HRP/DAB Detection IHC Kit, Abcam). Shortly, sections were de-paraffinised, rehydrated and upon blocking of unspecific binding, incubated with primary antibodies for 60min at room temperature. After incubation with polyclonal secondary antibody, detection of target antigens was performed via streptavidin-peroxidase-mediated colour reaction of AEC or DAB. The following primary antibodies were used: cytokeratin-20 (CK-20; Dako), MCPyV-L (Santa Cruz, 1:200, Ki-67 (Dako, 1:80), synaptophysin (Pierce Thermo Scientific, 1:80), chromogranin-A (Pierce Thermo Scientific, 1:500), Images were taken on an Olympus BX53.

**Supplementary References**

[S1] E.I. Deryugina, J.P. Quigley. *Methods Enzymol*, 2008, 444, 21.

[S2] O. Martinho, R. Silva-Oliveira, V. Miranda-Goncalves, C. Clara, J.R. Almeida, A.L. Carvalho, J.T. Barata, R.M. Reis. *Transl Oncol*, 2013, 6, 187.

[S3] E.C. Jensen. *Anat Rec (Hoboken)*, 2013, 296, 378.
