# Supplementary material for: A short‐term in vivo model for Merkel Cell Carcinoma
Source: Exp Dermatol. 2018 Mar 26;27(6):684–7. doi: 10.1111/exd.13529 (PMC6175323; doi:10.1111/exd.13529)
Supplement: Supplementary file 5 [file EXD-27-684-s005.docx]

**Supplementary Figure Legends**

**FIGURE S1** Schematic workflow of *ex ovo* CAM assay. Fertilized eggs were incubated for 3 days, the egg shell was then cracked into plastic dishes, following further incubation for 7 days. MCC cells were applied on vascular branches of the CAM and incubated for 3-7 days. The CAM with the attached grafts was excised, followed by FFPE-tissue embedding and sectioning. The tumour morphology was analysed by histology and immunostaining.

**FIGURE S2** Photo-documentation of growth behaviour of xenografted MCC cell lines. (A) MKL-1 (upper panel), PeTa (middle panel) and WaGa (lower panel) were monitored for 5 days upon engraftment. Bars equal 1 mm. (B) Tumour area per CAM was measured using Image J software. (C) Angiogenesis was measured by counting macroscopic blood vessels (MBV) manually. Results were plotted as mean±SD using GraphPad prism software. (N=6 tumours). One-way ANOVA was used for statistical analysis.

**FIGURE S3** Immunohistochemical characterizations of xenografted MCC cell lines with neuroendocrine specific marker. (A-F) All MCC cell lines express the neuroendocrine tumour specific markers synaptophysin (p38) and chromogranin A (100x and 400x magnification, scale bar = 100µm and 20μm respectively).
